# UBE2S emerges as a key driver in an NK cell–based prognostic model for clear cell renal cell carcinoma

**DOI:** 10.1371/journal.pone.0344925

**Published:** 2026-05-14

**Authors:** Kang Leng, Yiheng Zhou, Jian Zhao, Xiaoguang Wang, Hua Song

**Affiliations:** 1 Department of Urology, The 960th Hospital of the PLA Joint Logistics Support Force, Jinan, China; 2 Department of Intensive Care Unit, Jinan Fourth People’s Hospital Affiliated to Shandong Second Medical University, Jinan, China; Ondokuz Mayis University Faculty of Medicine: Ondokuz Mayis Universitesi Tip Fakultesi, TÜRKIYE

## Abstract

**Background:**

Clear cell renal cell carcinoma (ccRCC) is highly heterogeneous, and robust biomarkers for risk stratification and therapeutic guidance remain limited.

**Methods:**

We integrated single-cell RNA sequencing (scRNA-seq) of tumor and adjacent tissues with multi-cohort transcriptomic validation. High-dimensional weighted gene co-expression network analysis (hdWGCNA) was applied to identify pathogenic immune subsets and candidate genes. Prognostic modeling was performed using CoxBoost, and UBE2S function was validated by in vitro knockdown assays.

**Results:**

scRNA-seq revealed extensive remodeling of the tumor microenvironment, highlighting NK cell subpopulations with strong intercellular signaling. hdWGCNA identified 12 core genes enriched in protein processing and MAPK pathways, with UBE2S emerging as the top driver. A CoxBoost-based 12-gene signature demonstrated robust predictive accuracy across independent datasets. Functionally, UBE2S knockdown suppressed ccRCC cell proliferation and migration, while immune correlation analyses linked UBE2S to altered tumor immunogenicity and genomic stability.

**Conclusions:**

Our study identifies NK cell subsets and UBE2S as key contributors to ccRCC progression and establishes a clinically relevant 12-gene prognostic model, offering potential targets for precision therapy.

## Introduction

Clear cell renal cell carcinoma (ccRCC) is the most common histological subtype of kidney cancer, accounting for approximately 70–80% of all cases [1,2]. Its pathogenesis is primarily associated with mutations in the VHL gene and dysregulation of the hypoxia-inducible factor (HIF) signaling pathway [3–5]. Known risk factors include smoking, obesity, and hypertension [6]. Patients often remain asymptomatic in early stages, but may later present with classic symptoms such as hematuria, flank pain, and an abdominal mass [7,8]. Current treatment strategies primarily involve surgical resection for localized diseases, while advanced cases are managed with targeted therapies (e.g., sunitinib, pembrolizumab) and immunotherapy [9–12]. However, these approaches are frequently limited by drug resistance, significant individual variability in response, and unsatisfactory overall prognosis [13,14]. Therefore, there is an urgent need to develop accurate, reliable, and clinically applicable biomarkers and/or prognostic models to improve therapeutic decision-making and outcomes in ccRCC [15–17].

Single-cell RNA sequencing (scRNA-seq) is a high-throughput technology that enables gene expression profiling at the single-cell level, providing unprecedented resolution for dissecting cellular heterogeneity and microenvironment composition [18,19]. Recent applications of scRNA-seq in ccRCC have greatly advanced our understanding of the tumor microenvironment (TME) [20,21]. The ccRCC TME is a complex ecosystem comprising diverse cell types—including tumor cells, immune cells, fibroblasts, and endothelial cells—that collectively influence tumor initiation, progression, and therapy response [22,23]. By integrating scRNA-seq with bulk transcriptomics and other multi-omics data, researchers have identified key biological targets within the TME that are implicated in ccRCC malignancy and immune evasion, such as specific T-cell subpopulations and tumor-associated macrophages (TAMs) [24–26]. These findings offer novel insights into the mechanistic underpinnings of ccRCC.

Although recent single-cell RNA sequencing (scRNA-seq) studies have provided an overview of clear cell renal cell carcinoma (ccRCC), the specific contributions of NK cell subsets to tumor microenvironment (TME) heterogeneity and their synergistic interactions with ubiquitin-proteasome system components remain understudied. Most existing studies have primarily focused on T cell exhaustion or macrophage polarization, often overlooking the critical role of NK cell plasticity. In this study, we employed a novel approach combining high-dimensional weighted gene co-expression network analysis (hdWGCNA) with single-cell transcriptomics to precisely identify pathogenic NK cell subpopulations. Unlike traditional clustering methods, hdWGCNA detects robust co-expression modules in sparse single-cell data. We found that UBE2S is not only a differentially expressed gene but also a core driver within a key NK cell module associated with ubiquitin-proteasome dysfunction. We further constructed a robust prognostic model and, crucially, validated the mechanistic link between UBE2S and malignant phenotypes in vitro, providing a theoretical basis for targeting the ubiquitin-proteasome axis in ccRCC immunotherapy.

## Materials and methods

### Public data sources acquisition and pre-process

The single-cell sequencing data analyzed in this study were obtained from the GEO database under accession number GSE210038. This dataset comprises single-cell RNA sequencing profiles derived from seven tumor samples and two adjacent normal tissue samples collected from patients with clear cell renal cell carcinoma (ccRCC). In addition, bulk RNA-seq data for kidney renal clear cell carcinoma (KIRC) were downloaded from the UCSC Xena platform (https://xena.ucsc.edu/), which originate from The Cancer Genome Atlas (TCGA) cohort, along with the corresponding clinical survival data for prognostic analyses. Furthermore, datasets GSE167573 and E-MTAB-1980 were incorporated as independent external validation cohorts. Briefly, the GSE167573 dataset consists of bulk RNA-seq data from patients in China, processed with high-quality read counts. Similarly, the E-MTAB-1980 dataset, sourced from the University of Tokyo, Japan, comprises microarray profiles that passed strict quality control thresholds prior to validation analysis.

### Single-cell RNA-Seq processing

Single-cell RNA-seq data for ccRCC were analyzed in R using the Seurat package (v4.3.0) [27]. To ensure data quality, strict filtering criteria were applied. Cells with gene counts between 200 and 4000 and mitochondrial gene content <20% were retained. Doublet detection and removal were performed using the DoubletFinder package to eliminate potential cellular artifacts. The Harmony algorithm was used to integrate datasets from different samples to correct batch effects and remove technical noise. The data were then normalized and scaled using the LogNormalize method. Principal component analysis (PCA) was conducted, and the top 15 principal components were selected for t-SNE and UMAP dimensionality reduction to visualize cellular heterogeneity [28].

### High-dimensional WGCNA analysis

High-dimensional weighted gene co-expression network analysis (hdWGCNA) was employed to identify NK cell–related key genes in clear cell renal cell carcinoma (ccRCC) samples [29]. To address the inherent sparsity of scRNA-seq data, we constructed a robust co-expression network using the metacell aggregation method provided by the hdWGCNA package. NK cell populations were extracted from scRNA-seq data, followed by the construction of gene expression correlation matrices, weighted co-expression networks, and module detection. Based on the scale-free topology criterion (R² > 0.85), a soft threshold power of 6 was selected. Modules were detected using the GetModules function with a minimum module size of 50 genes. Module conservation was assessed to ensure the stability of the identified network. Module-feature relationships were analyzed to identify modules significantly associated with the metastatic group, and intra-module connectivity was evaluated based on hub genes within these key modules. The top 80 hub genes were considered representative of important NK cell-associated genes relevant to ccRCC progression.

### Cell communication analysis

The CellChat package was utilized as an analytical tool to investigate intercellular interactions and communication. Significant biological interactions among renal carcinoma cell populations were inferred using CellChat [30]. By integrating gene expression data with known information on signaling ligands, receptors, and cofactors, intercellular communication was modeled and analyzed, and the probabilities as well as statistical significance of these interactions were computed. The relationships and relative importance of different cell populations were visualized using circle plots and bubble plots.

### Pathway enrichment analysis

Differential expression of candidate feature genes between ccRCC and control samples was assessed using the limma package in R. Gene Ontology (GO) and Kyoto Encyclopedia of Genes and Genomes (KEGG) enrichment analyses were performed on these genes using the clusterProfiler package (v4.0) to determine their functional significance [31]. In addition, gene set variation analysis (GSVA) was applied to evaluate differences in KEGG pathway activity associated with the optimal feature genes. Statistical significance was defined at p < 0.05.

### Construction of prognostic models using machine learning

Multiple machine learning algorithms were employed to construct stable and accurate prognostic models. Data from TCGA, GSE167573, and E-MTAB-1980 were integrated to develop the optimal model. Consensus models were established based on algorithms including support vector machine (SVM), random forest (RF), and CoxBoost. The predictive performance of the models was evaluated using the area under the receiver operating characteristic curve (AUC of ROC). Prognostic differences were further assessed through Kaplan–Meier survival analysis, while the concordance index (C-index) was calculated to evaluate the accuracy of each model [32]. Collectively, these approaches were used to determine the most robust prognostic model.

### Prognostic analysis of UBE2S

The expression of the UBE2S gene was quantified from bulk RNA-seq data to construct high-risk and low-risk groups. To evaluate the expression patterns of these cell subpopulations within infiltrating tumors, bulk RNA-seq data were processed and single-sample gene set enrichment analysis (ssGSEA) was performed using the GSVA method. This approach enabled the quantitative assessment of the enrichment levels of these marker gene sets across diverse tumor samples. The enrichment results were then integrated with clinical survival data to assess prognostic differences between risk groups. For survival analyses, the survminer (version 0.4.9) and “survival” R packages were applied. Initially, the “surv_cutpoint” function was used to determine the optimal cut-off value for risk stratification. Based on this threshold, samples were categorized into high- and low-risk groups, and survival curves were generated using the “survfit” function.

### RT-qPCR analysis

Total RNA was extracted from cell lines using the Accurate Biology extraction kit (Hunan, China) according to the manufacturer’s instructions. RNA concentration and purity were assessed with a UV spectrophotometer, and 1 µg RNA was reverse-transcribed to cDNA (Accurate Biology kit). Quantitative real-time PCR was performed on a StepOnePlus™ system (Applied Biosystems). Gene-specific primers were: UBE2S forward 5′-CGATGGCATCAAGGTCTTTTCCC-3′ and reverse 5′-CAGCAGGAGTTTCATGCGGAAC-3′; GAPDH forward 5′-GGAGCGAGATCCCTCCAAAAT-3′ and reverse 5′-GGCTGTTGTCATACTTCTCATGG-3′. Melting-curve analysis was used to confirm amplicon specificity. Relative expression was calculated by the comparative ΔΔCt method with GAPDH as the internal control. All reactions were run in technical triplicate, and data are presented as mean ± SD.

### Western blot

Proteins were extracted with RIPA lysis buffer, quantified by the BCA assay, separated by SDS–PAGE, and transferred to PVDF membranes. After blocking with 5% skim milk, membranes were incubated overnight at 4 °C with primary antibodies against UBE2S (Proteintech, Cat No. 14115–1-AP, 1:1000) and GAPDH (Proteintech, Cat No. 10494–1-AP, 1:5000). After TBST washes, blots were incubated with HRP-conjugated goat anti-rabbit IgG (H + L) secondary antibody (Proteintech, SA00001–2, 1:3000) for 1 h at room temperature. Protein signals were developed using enhanced chemiluminescence on a chemiluminescence imaging system, and band intensities were quantified in ImageJ with normalization to GAPDH.

### Transwell assay

Cell invasion was assessed using Matrigel-coated Transwell chambers (Corning, USA). Briefly, 10,000 cells were seeded in the upper chamber containing serum-free medium, while medium supplemented with serum was added to the lower chamber as a chemoattractant. After incubation, invading cells were fixed with 4% paraformaldehyde, stained with 0.1% crystal violet, and counted in five randomly selected fields under an inverted microscope.

### Cell viability assay

Cell proliferation was measured using the CCK-8 kit (Beyotime, China). Cells were seeded into 96-well plates (1,000 cells/well) in triplicate. At the indicated time points, CCK-8 reagent was added and incubated at 37 °C for 30 min, and absorbance at 450 nm was measured using a microplate reader. Cell viability was expressed as mean ± SD from at least three independent experiments.

### Statistical analysis

Data processing, analysis, and visualization were carried out using R packages (version 4.30; https://www.bioconductor.org/) or Graphpad Prism software (version 8.0.1; https://www.graphpad.com/). For differential expression analysis, fold change >1.5 and adjusted P-value (false discovery rate, FDR) < 0.05 were used as criteria for statistical significance. Comparisons between two groups were performed using Student’s t-test, while comparisons among multiple groups were conducted using one-way ANOVA followed by Tukey’s post-hoc test. To control for potential confounding factors in the clinical data, multivariate Cox regression analysis was performed. P < 0.05 was considered statistically significant. Results are expressed as the mean ± standard error of the mean.

## Results

### Single-cell RNA-Seq analysis of ccRCC

**Fig 1:**
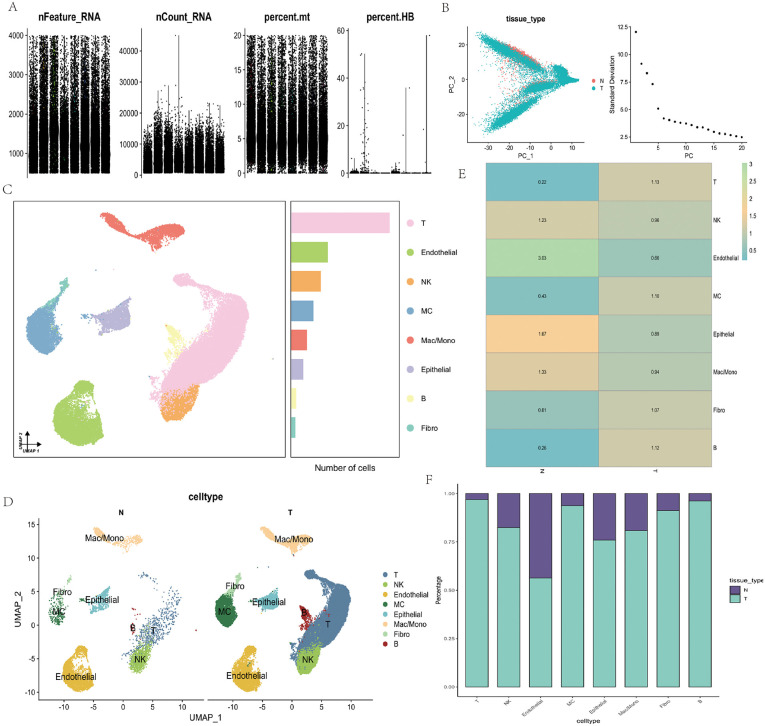
Single-cell RNA-Seq analysis of ccRCC. **(A)** Quality control metrics of single cells, including the number of detected genes per cell (nFeature_RNA), total UMI counts (nCount_RNA), percentage of mitochondrial transcripts (percent.mt), and hemoglobin gene expression ratio (percent.HB).(B) Principal component analysis (PCA) of single cells, colored by tissue type (normal vs. tumor), with an elbow plot showing the variance explained by the top principal components.(C) Uniform Manifold Approximation and Projection (UMAP) visualization of major cell clusters, annotated with distinct colors and corresponding cell-type proportions.(D) UMAP representation showing distribution of annotated cell types between tumor (T) and normal (N) samples.(E) Heatmap of average gene expression correlation across cell types, indicating the transcriptional similarity between clusters.(F) Bar plot showing the relative proportion of each cell type in tumor and normal tissues. All plots were generated using R software (version 4.2). Individual panels were assembled in Adobe Illustrator.

Single-cell transcriptomic analysis was performed on clear cell renal cell carcinoma (ccRCC) tissues and adjacent normal tissues to investigate cellular heterogeneity and identify potential therapeutic targets. Following quality control (Fig 1A), principal component analysis (PCA) (Fig 1B) revealed a distinct separation between tumor and normal tissues along the first two principal components, while elbow analysis indicated that the top 10 PCs effectively captured intercellular variation. Subsequent clustering using the UMAP algorithm (Fig 1C) identified eight major cell populations, including T cells, NK cells, B cells, macrophages/monocytes, mast cells, fibroblasts, endothelial cells, and epithelial cells, with notable differences in their relative abundances across subpopulations. When tissue origin was mapped onto the UMAP space (Fig 1D), most cell types were detected in both tumor and normal tissues, but with markedly different distributions. Specifically, T cells, macrophages/monocytes, and fibroblasts were enriched in tumor tissues, whereas endothelial cells and B cells were more abundant in normal tissues. Quantitative comparison of cell-type proportions (Fig 1E–F) further confirmed these differences.

Collectively, these findings indicate that the immune and stromal compartments of the ccRCC microenvironment undergo substantial remodeling, a process likely contributing to immune evasion and aberrant angiogenesis, and providing an essential cellular foundation for the identification of key molecular drivers and potential therapeutic targets.

### Identification of key cell populations by augur

**Fig 2:**
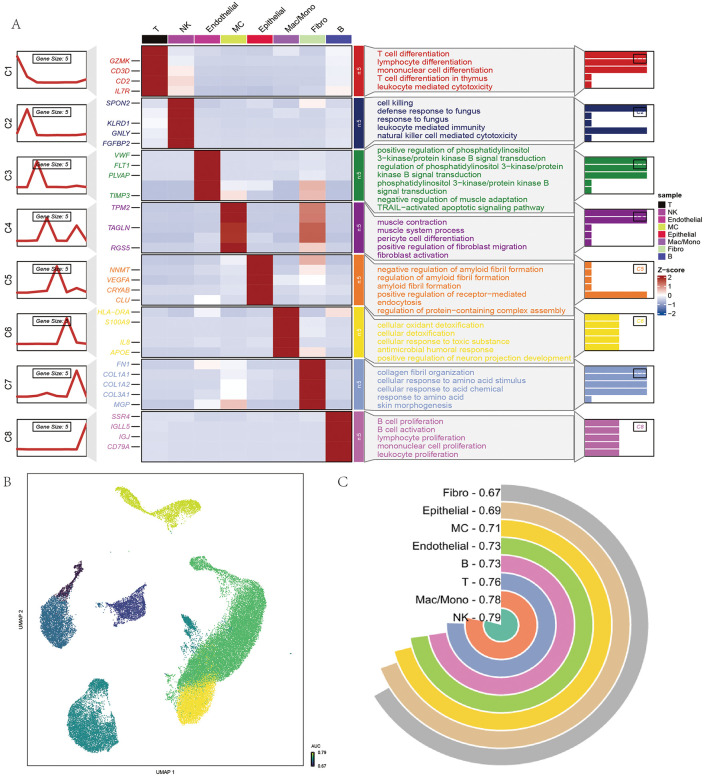
Identification of key cell populations by augur. **(A)** Heatmap of representative gene modules (C1–C8) across major cell types, with module eigengene expression patterns (left), top representative genes (middle), and enriched biological processes (right). **(B)** UMAP visualization of single cells colored by module activity scores, demonstrating cell type–specific enrichment of functional gene sets. **(C)** Ranking of cell types based on average module activity scores, with NK cells exhibiting the highest specificity (0.79), followed by Mac/Mono (0.78), T cells (0.76), and other cell lineages. All plots were generated using R software (version 4.2). Individual panels were assembled in Adobe Illustrator.

To further explore the heterogeneity of ccRCC, functional annotation of cell populations was conducted based on differential gene expression (Fig 2A). Distinct transcriptional signatures were identified: T cells were enriched in differentiation, cytotoxicity, and immune response pathways; NK cells in NK cell–mediated cytotoxicity and antifungal immunity; endothelial cells in angiogenesis and PI3K–AKT signaling; fibroblasts in extracellular matrix remodeling and migration; macrophages/monocytes in phagocytosis and inflammatory signaling; and B cells in activation and humoral immune regulation. These results indicate functional specialization among tumor microenvironment subtypes.

To pinpoint cell types most critical to ccRCC progression, the Augur algorithm was applied. UMAP visualization (Fig 2B) and quantitative analysis (Fig 2C) demonstrated that NK cells, macrophages/monocytes, and T cells had the greatest discriminatory power (AUC = 0.79, 0.78, and 0.76, respectively). Collectively, these findings suggest that immune cells—particularly NK cells—are central to ccRCC progression and immune microenvironment remodeling, highlighting them as promising candidates for mechanistic studies and therapeutic targeting.

### Dissection of critical NK cell subclusters

**Fig 3:**
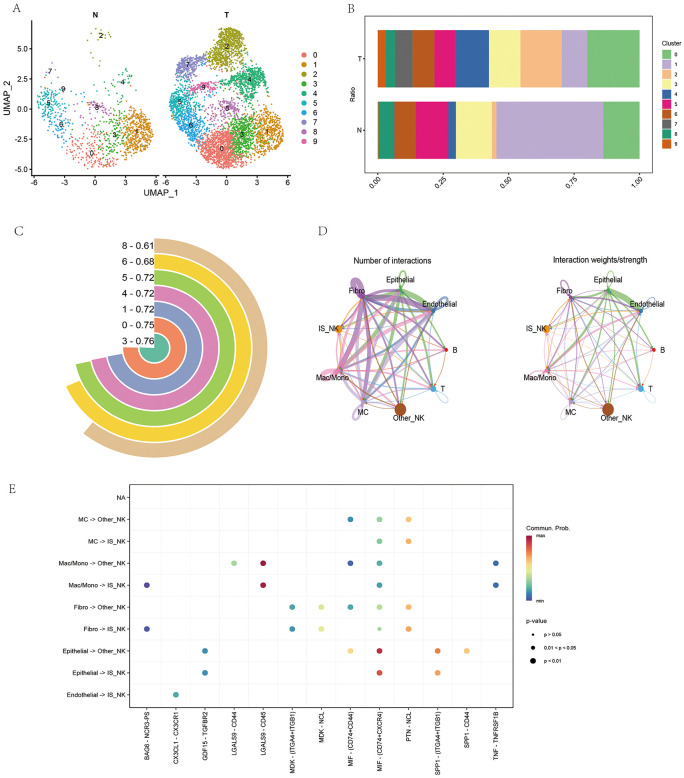
Dissection of critical NK cell subclusters. **(A)** UMAP visualization of tumor (T) and normal (N) tissues showing distinct NK cell subclusters (0–9). **(B)** Bar plot of the proportional distribution of NK cell subclusters between tumor and normal tissues, indicating tumor-specific enrichment of certain clusters. **(C)** Ranking of NK cell subclusters by functional activity scores, with subcluster 3 exhibiting the highest enrichment (0.76). **(D)** Network plots depicting intercellular communication among NK cell subclusters and other immune/stromal populations. The left panel shows the number of ligand–receptor interactions, while the right panel represents interaction strength. **(E)** Dot plot of significant ligand–receptor pairs mediating communication between NK subclusters and other cell types, with dot size indicating statistical significance and color representing communication probability. All plots were generated using R software (version 4.2). Individual panels were assembled in Adobe Illustrator.

To further investigate pathogenic components within NK cells, subclustering and functional assessment were performed on NK cells derived from tumor and normal tissues. UMAP analysis revealed multiple NK subpopulations with markedly different distributions between the two tissue types (Fig 3A). Several subclusters were significantly expanded in tumor tissues but less represented in normal samples, and quantitative comparisons confirmed these differences (Fig 3B). Augur analysis (Fig 3C) demonstrated that subclusters 3 and 0 had the highest discriminatory power between tumor and normal tissues (AUC = 0.76 and 0.75, respectively), suggesting their pivotal roles in ccRCC progression. These two subclusters were designated as IS_NK populations for subsequent analyses. Cell–cell communication analysis (Fig 3D) indicated extensive ligand–receptor interactions between IS_NK cells and macrophages/monocytes, fibroblasts, and endothelial cells, with both high frequency and strong signaling intensity. Detailed analysis of ligand–receptor pairs (Fig 3E) highlighted associations with immune- and tumor-related pathways, including MIF–CD74/ACKR4, MDK–NCL, SPP1–CD44, and TNF–TNFRSF1B.

Collectively, these results demonstrate that NK cells harbor functionally distinct subpopulations, with certain subsets remaining highly active in the tumor microenvironment. Through complex intercellular communication networks, these subpopulations likely contribute to immune regulation, tumor progression, and immune evasion in ccRCC.

### Signaling network analysis and pseudotime trajectory of NK cell subpopulations

**Fig 4:**
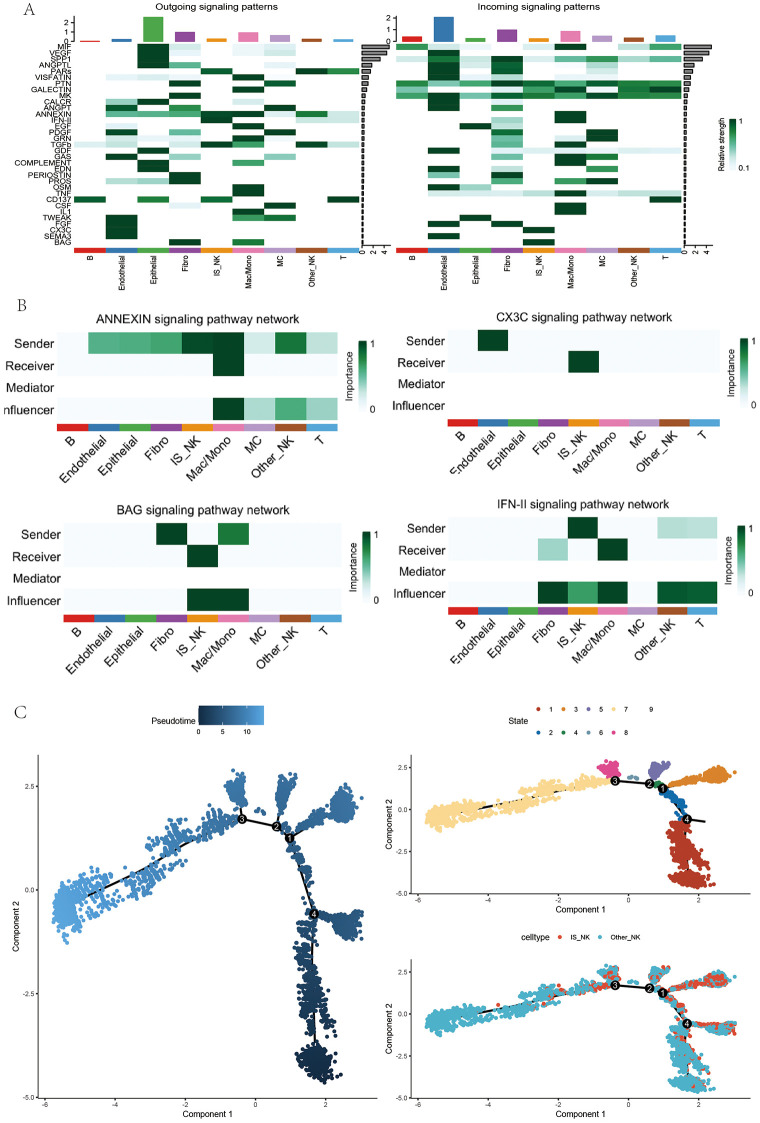
Signaling network analysis and pseudotime trajectory of NK cell subpopulations. **(A)** Heatmaps showing outgoing (left) and incoming (right) signaling patterns across different cell populations. The relative strength of each signaling pathway is depicted, highlighting major pathways involved in NK cell–mediated intercellular communication. **(B)** Detailed pathway-specific signaling networks, including ANNEXIN, CX3C, BAG, and IFN-II pathways. For each pathway, cell populations are annotated as senders, receivers, mediators, or influencers, demonstrating their roles in modulating NK cell signaling. **(C)** Pseudotime trajectory analysis of NK cell subpopulations. Left: trajectory plot colored by pseudotime progression. Middle: trajectory branches annotated by distinct cellular states. Right: trajectory colored by NK cell subtypes (IS_NK and Other_NK), suggesting differentiation dynamics within NK lineages. All plots were generated using R software (version 4.2). Individual panels were assembled in Adobe Illustrator.

To investigate the dynamic states and intercellular communication of NK cell subpopulations, a signaling network was first constructed across distinct subsets (Fig 4A). NK cells were positioned as central hubs for both sending and receiving signals across multiple pathways. Notably, IS_NK subpopulations displayed strong interactions with macrophages/monocytes, fibroblasts, and endothelial cells, particularly within immune-related pathways such as IFN-II, ANNEXIN, and CX3C, underscoring their active engagement in immune regulation. Moreover, the BAG signaling axis was prominently enriched between NK cells and tumor-associated fibroblasts, suggesting that NK cells may contribute to microenvironmental remodeling by modulating inflammation- and apoptosis-related signals (Fig 4B).

Further pseudotime trajectory analysis (Fig 4C) delineated the dynamic evolution of NK cell subsets during tumor progression. IS_NK and Other_NK populations occupied distinct branches of the trajectory, with IS_NK positioned at an early developmental state. These findings highlight the presence of specialized NK cell niches within the ccRCC microenvironment and suggest that specific subpopulations are poised to exert critical regulatory functions in tumor progression.

### Identification of key gene modules of NK cell by hd-WGCNA

**Fig 5:**
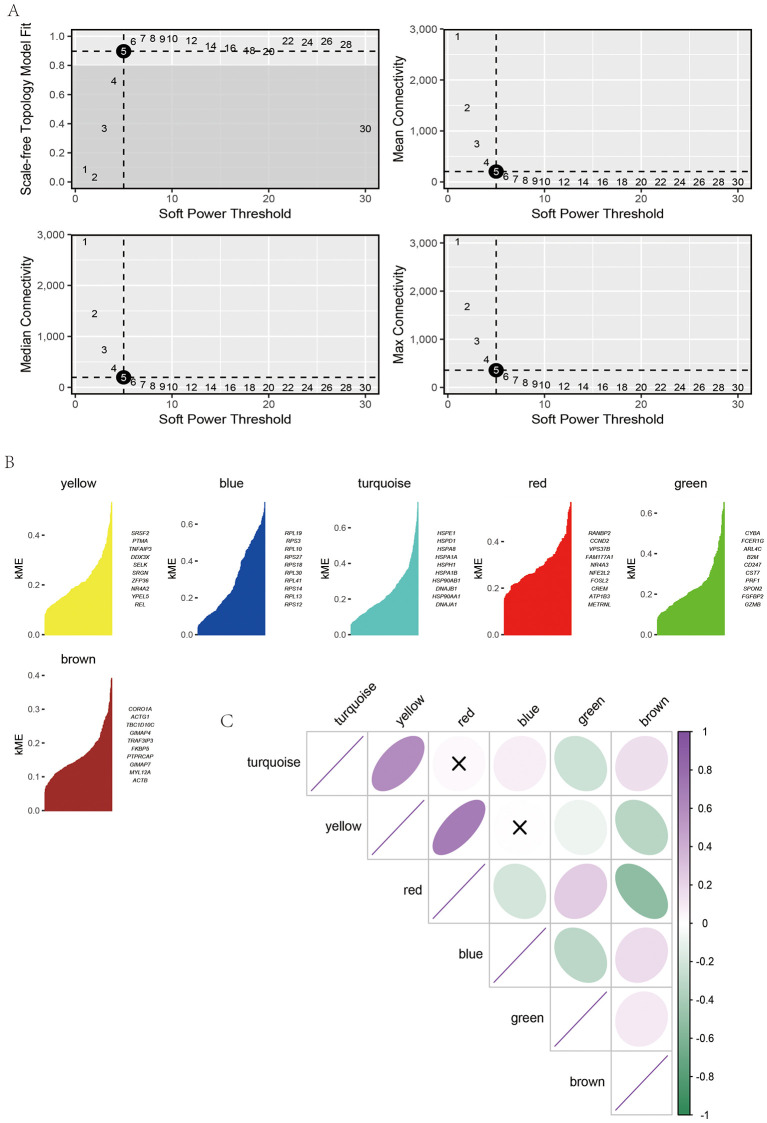
Identification of key gene modules of NK cell by hd-WGCNA. **(A)** Determination of the optimal soft-thresholding power for WGCNA. Plots show the scale-free topology model fit (top left), mean connectivity (top right), median connectivity (bottom left), and maximum connectivity (bottom right) across a range of soft-threshold powers. A soft-threshold power of 6 was selected to achieve a scale-free topology with balanced network connectivity. **(B)** Module membership (kME) distribution of key co-expression modules (yellow, blue, turquoise, red, green, and brown), with representative hub genes labeled for each module. **(C)** Correlation analysis among identified modules, presented as a module–module relationship matrix. All plots were generated using R software (version 4.2). Individual panels were assembled in Adobe Illustrator.

To further characterize the transcriptional regulatory architecture of NK cells, high-dimensional weighted gene co-expression network analysis (hdWGCNA) was performed. Topological fitting and connectivity assessments across multiple soft-threshold powers (Fig 5A) identified a threshold of 6, which preserved scale-free topology while maintaining reasonable gene connectivity. Under this parameter, NK cell transcriptomes were decomposed into several co-expression modules (Fig 5B), including yellow, blue, turquoise, red, green, and brown modules, each representing groups of genes with coordinated expression patterns and distinct hub genes. Correlation analysis among these modules (Fig 5C) revealed strong inter-module associations, suggesting functional coordination across transcriptional programs. Together, these findings indicate that NK cells in the ccRCC microenvironment are organized into modular co-expression networks that cooperatively regulate immune responses, stress adaptation, and metabolic activity. This modular architecture provides novel insights into the functional diversification of NK cells in tumor immunity.

### Module characterization of NK cell subpopulations

**Fig 6:**
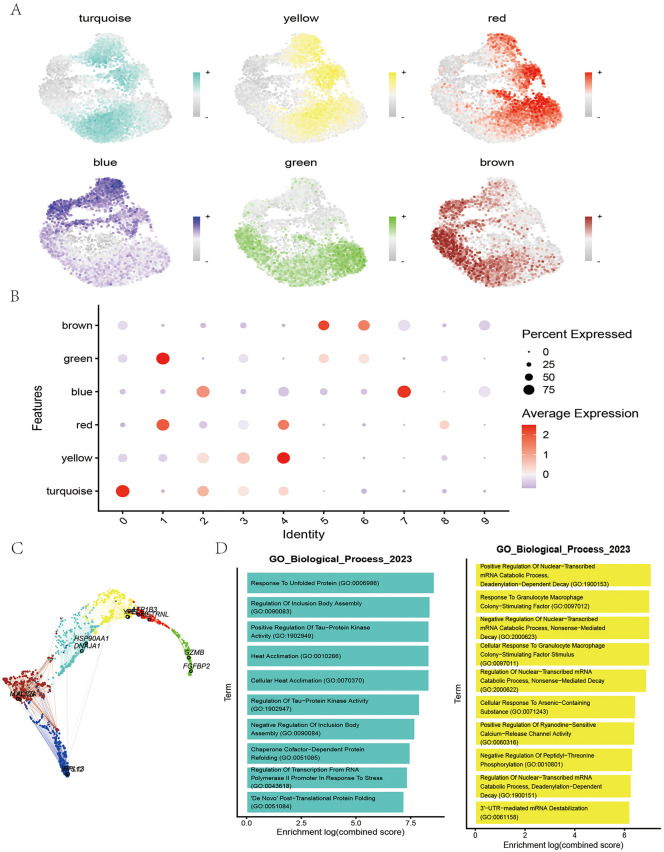
Module characterization of NK cell subpopulations. **(A)** The UMAP plot shows cell populations marked by different modules. **(B)** The bubble chart displays the average expression levels and expression percentages of modules in different cell populations. **(C)** The network diagram shows genes associated with specific cell populations. **(D)** GO biological process enrichment analysis. The bar chart presents significantly enriched biological processes related to different cell populations. All plots were generated using R software (version 4.2). Individual panels were assembled in Adobe Illustrator.

To define functional modules associated with NK cell subsets, eigengene modules were projected onto the NK cell single-cell transcriptomes, allowing assessment of their associations across subclusters (Fig 6A). Correlation scores were calculated for each subcluster–module pair and visualized as a heatmap. Consistent with earlier analyses identifying subclusters 0 and 3 as the most critical NK subsets, the heatmap revealed the strongest associations with the turquoise and yellow modules (Fig 6B), indicating that these modules represent key pathogenic programs in NK cells. To explore inter-module relationships, module genes were visualized in principal component space, where turquoise and yellow modules clustered closely together, confirming their functional relatedness (Fig 6C). hdWGCNA analysis thus highlighted subclusters 0 and 3 as pathogenic NK populations, with their pathogenic signatures concentrated in turquoise and yellow modules. GO enrichment of module genes further revealed that the turquoise module was enriched in unfolded protein response and inclusion body assembly, whereas the yellow module was associated with positive regulation of nuclear transcription, mRNA catabolic processes, deadenylation-dependent decay, and granulocyte–macrophage responses (Fig 6D). Together, these findings suggest that pathogenic NK subclusters are driven by dysregulation of transcriptional programs, protein assembly, and immune activation.

### Identification of core prognostic genes

**Fig 7:**
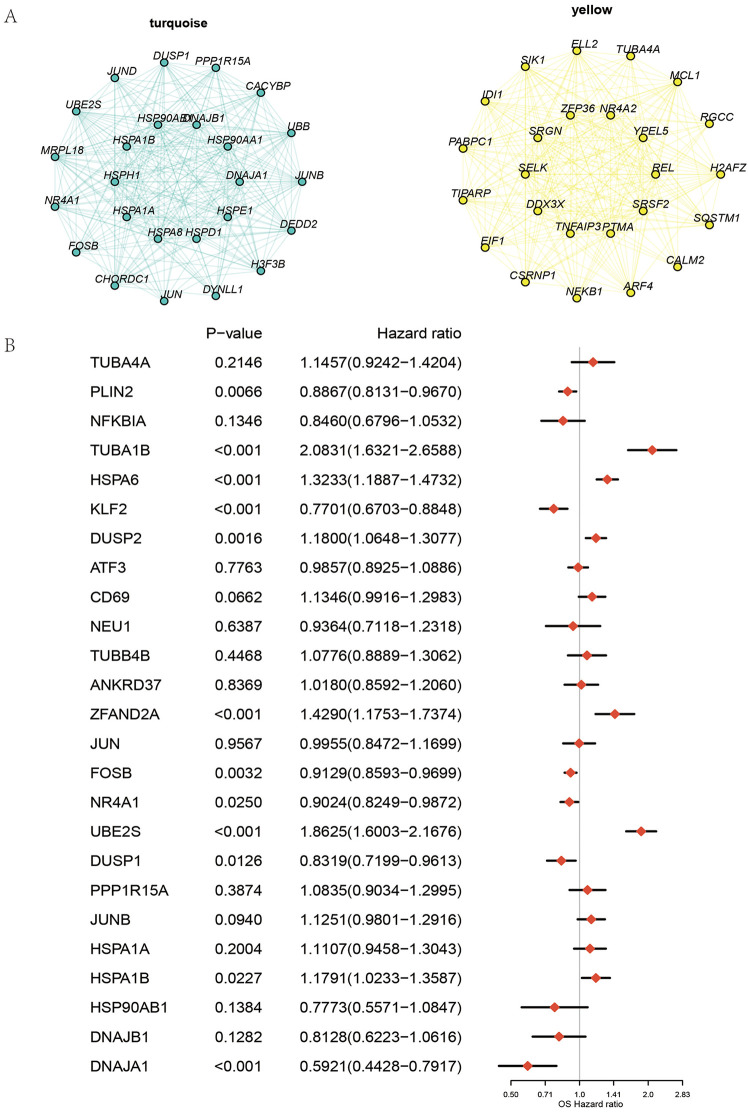
Identification of core prognostic genes. **(A)** The network diagram shows the interactions of differentially expressed genes in the turquoise and yellow cell populations. Nodes represent genes, and edges indicate interactions between genes. Genes in the turquoise and yellow cell populations are represented by nodes of different colors. **(B)** The forest plot illustrates the hazard ratios of significantly differentially expressed genes in univariate regression along with their 95% confidence intervals. All plots were generated using R software (version 4.2). Individual panels were assembled in Adobe Illustrator.

A series of single-cell analyses established NK cells as key pathogenic populations in ccRCC, with subclusters 0 and 3 playing central roles. Differential expression analysis between tumor and normal tissues identified transcriptional alterations unique to these subsets. Consistent with our hdWGCNA results, which highlighted the turquoise and yellow modules as critical pathogenic programs, we compiled the eigengenes of these modules to represent the functional signatures of NK cells (Fig 7A). Intersecting module eigengenes with the differential expression results yielded 25 candidate pathogenic genes.

To construct a robust prognostic model, univariate Cox regression was performed to evaluate their clinical relevance in ccRCC (Fig 7B). Using a threshold of p < 0.05, 12 core genes—including PLIN2, TUBA1B, HSPA6, KLF2, DUSP2, ZFAND2A, FOSB, NR4A1, UBE2S, DUSP1, HSPA1B, and DNAJA1—were identified as the most critical drivers for model development.

### Construction and evaluation of prognostic models

**Fig 8:**
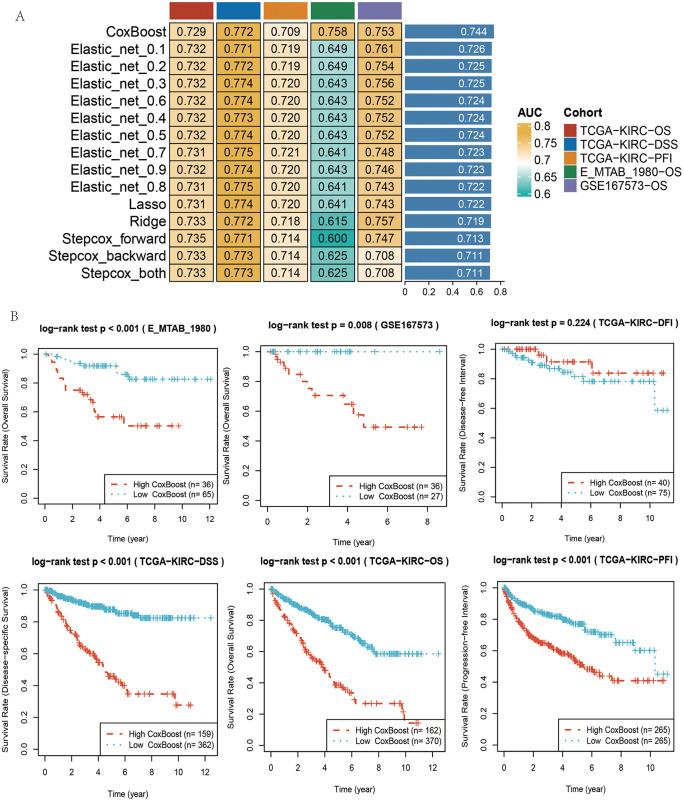
Construction and evaluation of prognostic models. **(A)** The heatmap shows the prediction performance of different models in four cohorts, using AUC (Area Under the Curve) as the metric. The models include CoxBoost, Elastic_net (with different parameter settings), Lasso, Ridge, Stepcox (forward and backward selection), and Stepcox (combination of both). The depth of color indicates the level of AUC values, with red representing higher AUC values and blue representing lower AUC values. The legend on the right shows the range of AUC values for different cohorts. **(B)** The survival curve plot displays the prediction performance of different models across six cohorts. Each subplot shows the survival rates over time for the high-risk group (red) and low-risk group (cyan). All plots were generated using R software (version 4.2). Individual panels were assembled in Adobe Illustrator.

To identify the optimal prognostic framework, models were fitted using a panel of machine learning algorithms, including logistic regression, elastic net (Enet), k-nearest neighbors (KNN), random forest (RF), support vector machine (SVM), multilayer perceptron (MLP), LightGBM, XGBoost, and decision tree (DT) (Fig 8A). Model performance was assessed across five survival endpoints: overall survival (OS), disease-specific survival (DSS), and progression-free interval (PFI) in the TCGA ccRCC cohort, as well as validation in the E-MTAB-1980 and GSE167573 datasets.

Consensus clustering of 12 key genes revealed that the CoxBoost algorithm achieved the most robust predictive performance, with an AUC of 0.744, indicating strong model stability. To further validate the clinical utility of the model, we conducted decision curve analysis (DCA), and the results showed that this prognostic model provided a higher net benefit compared to treating all patients or treating no patients (S1 Fig). In addition, the calibration curves for 1-year, 3-year, and 5-year survival rates showed excellent agreement between predicted probabilities and observed outcomes (S2 Fig). Our model’s C-index was calculated to be 0.76, outperforming traditional clinical variables. Survival analysis further demonstrated clear stratification of patients into high- and low-risk groups, with the high-risk group exhibiting significantly poorer outcomes compared to the low-risk group (Fig 8B).

Together, these findings establish a 12-gene CoxBoost-based prognostic model capable of reliably predicting patient outcomes in ccRCC, underscoring its potential clinical utility for risk stratification and precision prognostication.

### Dissecting the molecular characterization of model genes

**Fig 9:**
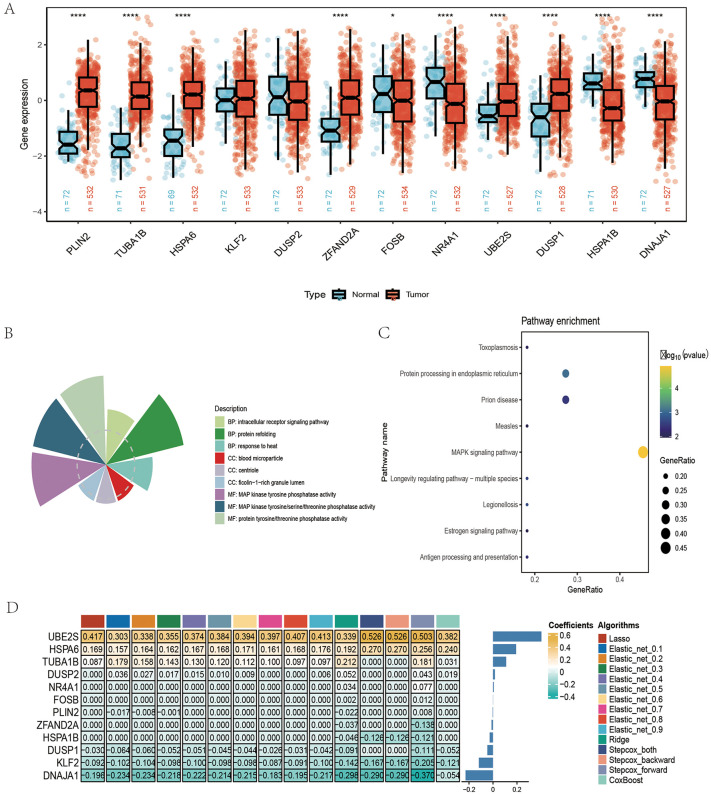
Dissecting the molecular characterization of model genes. **(A)** The box plot shows the expression level differences of multiple genes between normal tissue and tumor tissue. **(B)** The pie chart displays the results of gene enrichment analysis, categorized by biological process. **(C)** The bubble chart presents the results of gene set enrichment analysis (GSEA), categorized by signaling pathway. **(D)** The heatmap illustrates the coefficients of genes across different algorithm models. All plots were generated using R software (version 4.2). Individual panels were assembled in Adobe Illustrator.

Expression analysis revealed marked differences in most genes between ccRCC and normal tissues (Fig 9A). Pathway enrichment analysis was then performed to identify critical regulatory pathways. GO enrichment demonstrated significant involvement in MAPK enzymatic activity (Fig 9B), while KEGG analysis highlighted enrichment in the MAPK signaling cascade and protein processing pathways (Fig 9C). These findings suggest that the modeling genes primarily contribute to ccRCC pathogenesis through modulation of protein processing within the MAPK pathway, thereby altering enzymatic activity. To identify the most influential gene in our prognostic model, we examined the contribution of each feature gene to model performance. Notably, UBE2S consistently emerged as the top contributor across different modeling approaches (Fig 9D). This result indicates that UBE2S is a critical driver in the prognostic signature and may serve as a key dependency in ccRCC progression.

### Prognostic and therapeutic implications of UBE2S

**Fig 10:**
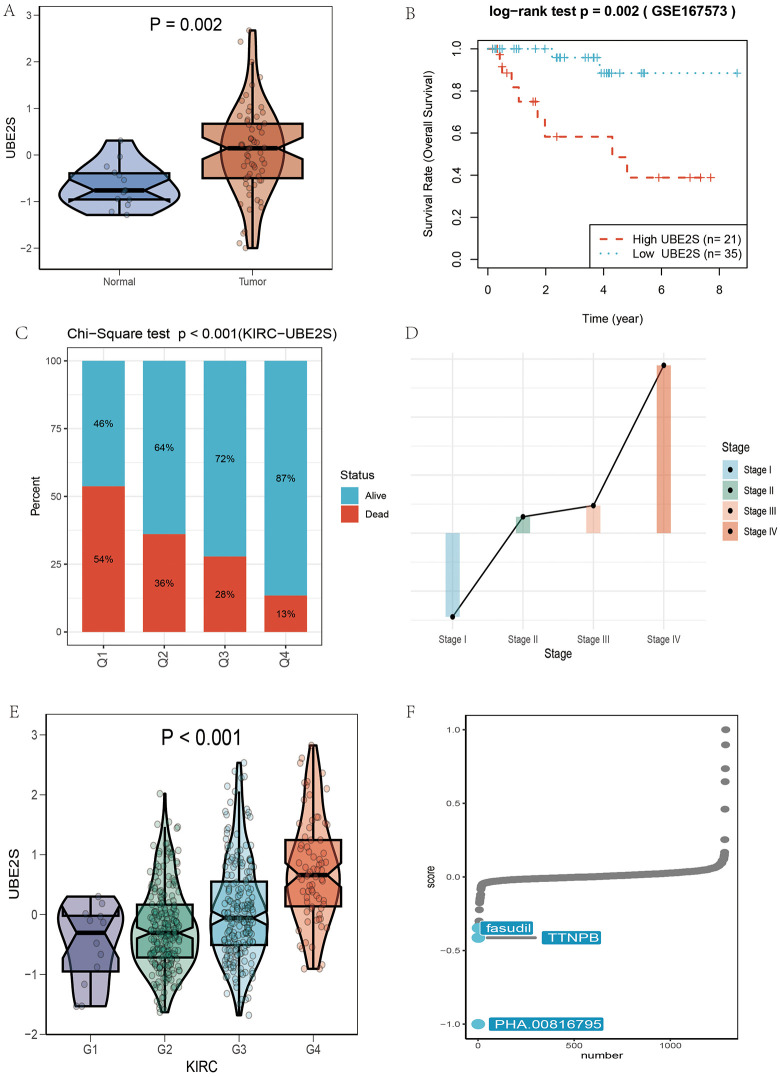
Prognostic and therapeutic implications of UBE2S. **(A)** The violin plot shows the expression distribution of the UBE2S gene in normal tissues and tumor tissues. **(B)** The survival curve illustrates the overall survival rate of the high UBE2S expression group (red, n = 21) and the low UBE2S expression group (cyan, n = 35) in the GSE167573 cohort. **(C)** The bar chart displays the distribution of patient survival status across different clinical stages (Q1 to Q4) in the KIRC-UBE2S dataset. **(D)** The stacked bar chart shows the distribution of patient survival status across different clinical stages (Stage I to Stage **IV)**. **(E)** The violin plot illustrates the distribution of UBE2S gene expression across different clinical stages (G1 to G4) in the KIRC dataset. **(F)** Drug sensitivity analysis shows the potential therapeutic drugs for the gene. All plots were generated using R software (version 4.2). Individual panels were assembled in Adobe Illustrator.

Expression analysis in the GEO validation cohort revealed that UBE2S was significantly upregulated in tumor samples compared with normal tissues (Fig 10A). Survival analysis further demonstrated that UBE2S expression stratified patient outcomes (Fig 10B). When patients were divided into quartiles based on expression levels, those in the highest quartile (Q1) exhibited a markedly higher mortality rate than the other groups (Fig 10C), indicating that high UBE2S expression is associated with poor prognosis. Analysis across tumor stages (Fig 10D) showed that UBE2S expression increased progressively from stage I to stage IV, while comparison across tumor grades (Fig 10E) revealed higher expression in advanced grades (G3 and high grade) relative to lower grades (G1–G2). These findings suggest that UBE2S may drive tumor progression in KIRC, and its expression correlates with disease aggressiveness. To explore potential therapeutic strategies, cMAP analysis was performed. Notably, PHA.00816795 was identified as a candidate compound capable of reversing the molecular signatures associated with aberrant UBE2S expression, thereby potentially mitigating its oncogenic effects (Fig 10F).

### Immune regulatory correlates of UBE2S

**Fig 11:**
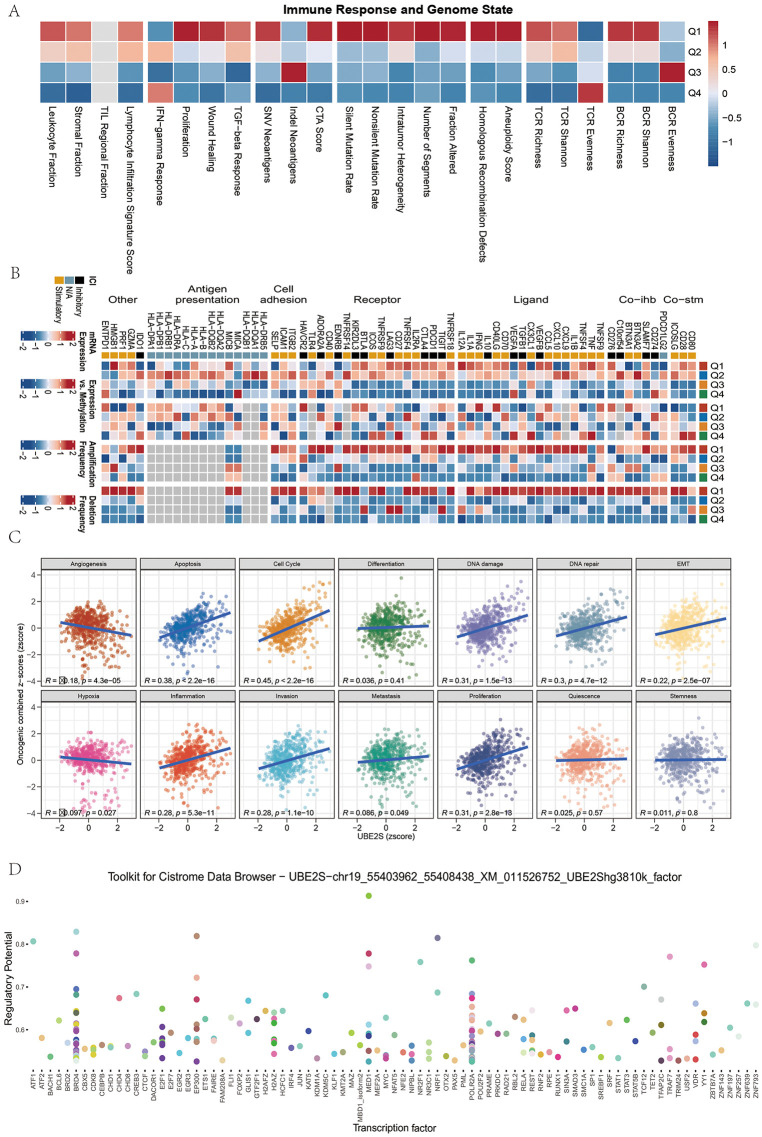
Immune regulatory correlates of UBE2S. **(A)** The heatmap shows the distribution of different immune response and genome status indicators across four different groups (Q1 to Q4).(B) The heatmap displays the expression of different immune checkpoints, antigen presentation, cell adhesion, receptors, and ligands in the four different groups.(C) The scatter plot illustrates the correlation between UBE2S expression and the expression of related genes in different biological processes (such as angiogenesis, apoptosis, cell cycle, differentiation, DNA damage, DNA repair, and epithelial-mesenchymal transition).(D) The scatter plot shows the correlation between different transcription factors from the Cistrome data browser and UBE2S gene expression. Each point represents a sample, with the color indicating the size and direction of the correlation coefficient; green represents a positive correlation, and purple represents a negative correlation. All plots were generated using R software (version 4.2). Individual panels were assembled in Adobe Illustrator.

Immune regulatory molecules are critical determinants of cancer immunotherapy, and numerous agonists and antagonists are currently being evaluated in clinical oncology. To explore the role of UBE2S, patients were stratified into quartiles (Q1–Q4) based on expression levels. High-expression groups exhibited stronger responses in immunogenicity and DNA damage–related scores (Fig 11A–B), indicating that elevated UBE2S expression may reshape the immune landscape of ccRCC. Correlation analyses further revealed that UBE2S expression was positively associated with cell-cycle activity, DNA damage, and DNA repair programs in KIRC (Fig 11C), suggesting that UBE2S actively participates in tumor cell proliferation and genomic instability. Finally, transcription factor enrichment analysis identified BRD4 and POLR2A as potential upstream regulators of UBE2S (Fig 11D), highlighting putative regulatory mechanisms driving its expression.

### Experimental validation of UBE2S in ccRCC

**Fig 12:**
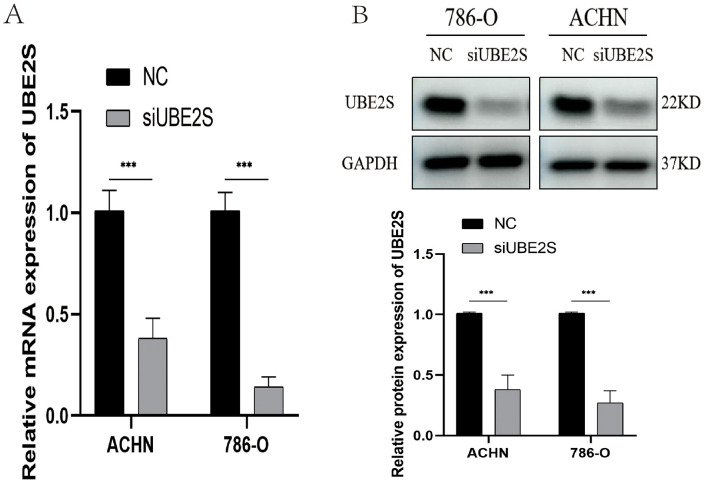
Knockdown validation of siUBE2S in ccRCC. **(A)** The bar chart shows the relative mRNA expression levels of the UBE2S gene in ACHN and 786-O cell lines. The black bars represent the control group (NC), while the gray bars represent the UBE2S gene silencing group (siUBE2S). **(B)** Upper panel: Western blot analysis shows the expression of UBE2S protein in 786-O and ACHN cell lines. Lower panel: The bar chart displays the relative expression levels of UBE2S protein.

**Fig 13:**
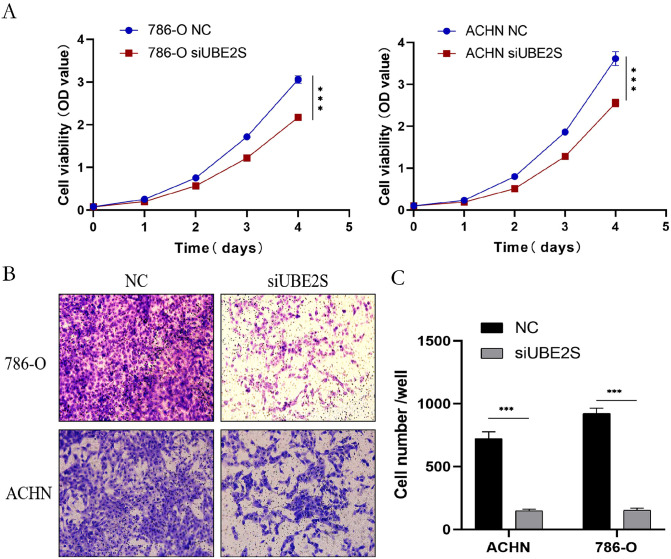
Experimental validation of UBE2S in ccRCC. **(A)** The cell viability assay results show the impact of UBE2S gene silencing (siUBE2S) on cell viability in the 786-O and ACHN cell lines. **(B)** The cell proliferation assay results illustrate the cell proliferation in the 786-O and ACHN cell lines for the control group (NC) and the UBE2S gene silencing group (siUBE2S). **(C)** The bar graph shows the number of cells in the control group (NC) and the UBE2S gene silencing group (siUBE2S) in the ACHN and 786-O cell lines.

To investigate the potential role of UBE2S in ccRCC, siRNA-mediated knockdown was performed in 786-O and ACHN cells. Efficient silencing was confirmed by qPCR and Western blot (Fig 12A and B). Functional assays demonstrated that UBE2S depletion markedly suppressed cell proliferation, as shown by the CCK-8 assay (Fig 13A), and significantly impaired cell migration, as revealed by the Transwell assay (Fig 13B). Together, these findings underscore the critical role of UBE2S in sustaining the malignant phenotype of ccRCC cells.

## Discussion

This study aims to investigate the role of pathogenic cell subsets and their marker genes in the pathogenesis and progression of ccRCC. Through systematic single-cell transcriptomic analysis and clinical validation, we revealed the critical role of a specific NK cell subset and its key gene, UBE2S, in ccRCC, and preliminarily elucidated its underlying mechanisms.

First, key pathogenic cell subsets were identified in both normal and ccRCC tissues, and marker genes for these subsets were screened using the hdWGCNA method. Integrated clinical correlation analysis across multiple transcriptomic datasets further identified 12 key genes, suggesting their potential role as core drivers of dysfunctional mitophagy in ccRCC. Thus, this study confirms that NK cell subsets play an important role in ccRCC through these key genes. Functional enrichment analysis indicated that these genes are significantly involved in biological processes such as protein validation and transcriptional protein regulation. Existing literature suggests that ccRCC development is closely associated with disrupted protein homeostasis, particularly aberrant regulation of protein synthesis and degradation, which can promote tumor progression [33,34]. Based on this, we hypothesize that these key genes may contribute to ccRCC development by regulating protein biogenesis processes.

Subsequently, a prognostic prediction model was constructed using a set of 12 genes—PLIN2, TUBA1B, HSPA6, KLF2, DUSP2, ZFAND2A, FOSB, NR4A1, UBE2S, DUSP1, HSPA1B, and DNAJA1—from which UBE2S was identified as the most critical gene. Previous studies have shown that UBE2S, as an E2 ubiquitin-conjugating enzyme, is involved in cell cycle regulation and tumor progression in various cancers [35]; however, its role in ccRCC remains insufficiently studied. This study is the first to propose UBE2S as a key gene in ccRCC. Further functional analysis revealed that UBE2S is significantly associated with protein regulatory processes and cancer-related signaling pathways. Given that aberrant protein homeostasis has been reported to play an important role in ccRCC [36], and UBE2S is a component of the ubiquitin-proteasome system, it may influence tumorigenesis by modulating the stability of key proteins [35,36]. Therefore, we speculate that UBE2S may contribute to ccRCC progression through ubiquitination pathways and increase the risk of tumor development.

Despite the promising results, this study still has several limitations that need to be acknowledged. First, the sample size of the single-cell sequencing cohort used in this study is relatively small (seven tumor samples and two adjacent normal tissues), which may not fully capture the extensive inter-patient heterogeneity of ccRCC. Second, although we have validated the effects of UBE2S on cell phenotype and MAPK signaling in vitro, the lack of in vivo animal experiments limits our understanding of its role in a systemic physiological context. Third, the specific molecular mechanisms by which UBE2S regulates MAPK phosphorylation—whether through direct ubiquitination of pathway components or upstream regulators—remain to be elucidated by future structural and biochemical studies. Finally, our study lacks validation of UBE2S at the protein level within a local clinical cohort. As elegantly demonstrated by Du G. et al. [37], analyzing target proteins via immunohistochemistry (IHC) from local patient samples provides robust prognostic value. Future studies should validate UBE2S expression using IHC, as protein-level evaluation is much more feasible and practical for routine clinical application than mRNA-level investigations.

## Supporting information

S1 Data S1 File. R script for raw data preprocessing and quality control. S2 File. R script for differential expression analysis. S3 File. R script for functional enrichment analysis. S4 File. R script for survival analysis and prognostic model construction. S5 File. R script for data visualization and figure generation.
(ZIP)

S1 Fig Decision Curve Analysis (DCA) of the prognostic model. Evaluation of the clinical net benefit of the 12-gene signature for predicting 1-, 3-, and 5-year overall survival (OS) in the TCGA-KIRC cohort. The y-axis measures the net benefit, and the x-axis represents the threshold probability. The red solid line represents the CoxBoost prognostic model. The gray dashed line represents the strategy of assuming all patients will have the event (Treat All), while the black solid linerepresents the strategy of assuming no patients will have the event (Treat None). The model demonstrates a higher net benefit across a wide range of threshold probabilities compared to the default strategies at 1, 3, and 5 years.
(DOCX)

S2 Fig Calibration curves of the prognostic model. The plots illustrate the calibration of the model for predicting 1-, 3-, and 5-year overall survival (OS) in the TCGA-KIRC cohort. The x-axis represents the nomogram-predicted survival probability, and the y-axis represents the actual observed survival proportion. The gray diagonal dashed line represents the ideal prediction (perfect calibration). The close alignment of the model’s performance lines with the diagonal indicates robust predictive accuracy.
(DOCX)
