# Supplementary material for: UBE2S emerges as a key driver in an NK cell–based prognostic model for clear cell renal cell carcinoma
Source: PLoS One. 2026 May 14;21(5):e0344925. doi: 10.1371/journal.pone.0344925 (PMC13175336; doi:10.1371/journal.pone.0344925)
Supplement: S1 Fig — Evaluation of the clinical net benefit of the 12-gene signature for predicting 1-, 3-, and 5-year overall survival (OS) in the TCGA-KIRC cohort. The y-axis measures the net benefit, and the x-axis represents the threshold probability. The red solid line represents the CoxBoost prognostic model. The gray dashed line represents the strategy of assuming all patients will have the event (Treat All), while the black solid linerepresents the strategy of assuming no patients will have the event (Treat None). The model demonstrates a higher net benefit across a wide range of threshold probabilities compared to the default strategies at 1, 3, and 5 years. (DOCX) [file pone.0344925.s002.docx]

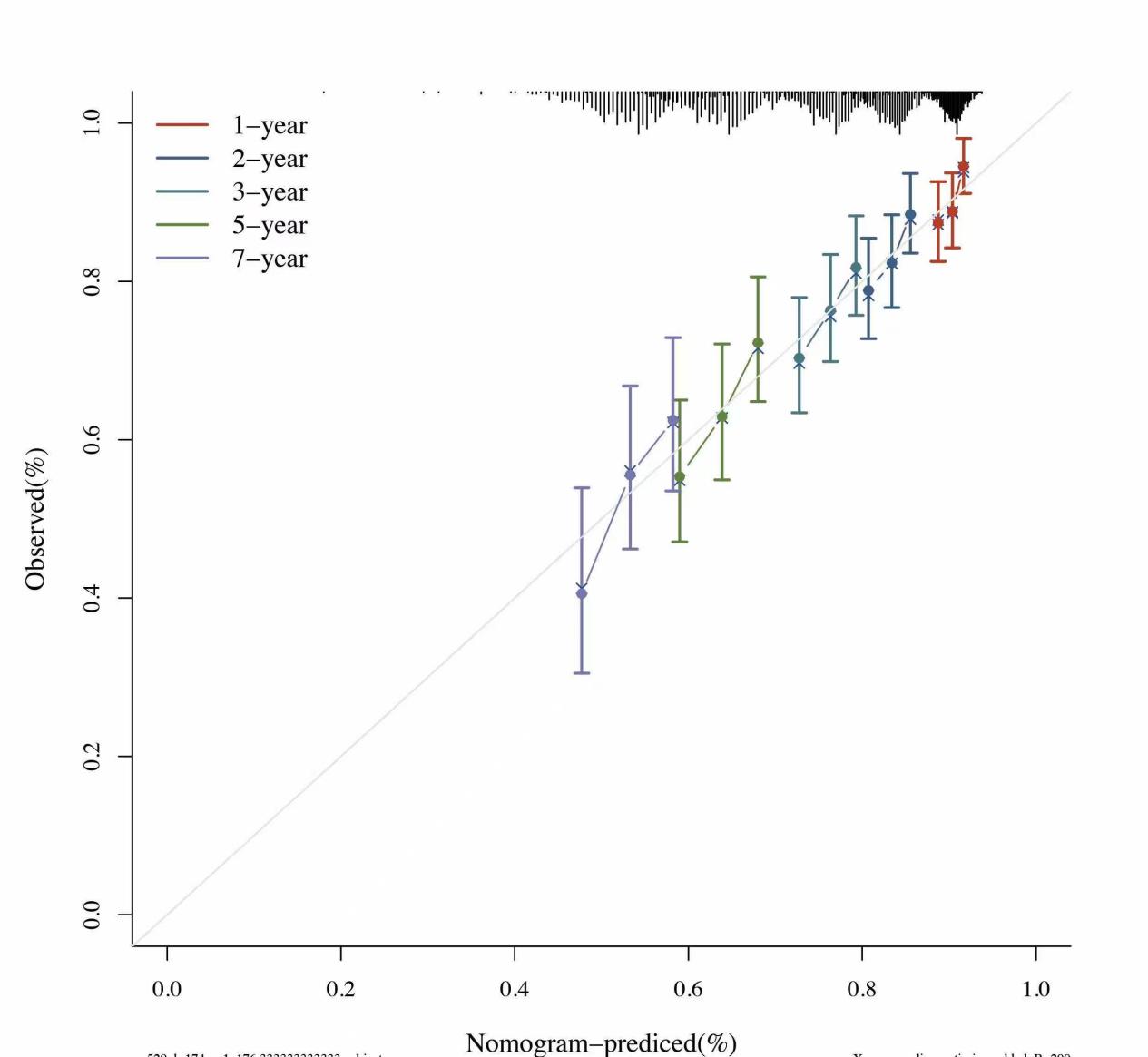


S1 Fig: **[Decision Curve Analysis (DCA) of the prognostic model.]**

[Evaluation of the clinical net benefit of the 12-gene signature for predicting 1-, 3-, and 5-year overall survival (OS) in the TCGA-KIRC cohort. The y-axis measures the net benefit, and the x-axis represents the threshold probability. The red solid line represents the CoxBoost prognostic model. The gray dashed line represents the strategy of assuming all patients will have the event (Treat All), while the black solid linerepresents the strategy of assuming no patients will have the event (Treat None). The model demonstrates a higher net benefit across a wide range of threshold probabilities compared to the default strategies at 1, 3, and 5 years.]
