# Supplementary material for: UBE2S emerges as a key driver in an NK cell–based prognostic model for clear cell renal cell carcinoma
Source: PLoS One. 2026 May 14;21(5):e0344925. doi: 10.1371/journal.pone.0344925 (PMC13175336; doi:10.1371/journal.pone.0344925)
Supplement: S2 Fig — The plots illustrate the calibration of the model for predicting 1-, 3-, and 5-year overall survival (OS) in the TCGA-KIRC cohort. The x-axis represents the nomogram-predicted survival probability, and the y-axis represents the actual observed survival proportion. The gray diagonal dashed line represents the ideal prediction (perfect calibration). The close alignment of the model’s performance lines with the diagonal indicates robust predictive accuracy. (DOCX) [file pone.0344925.s003.docx]

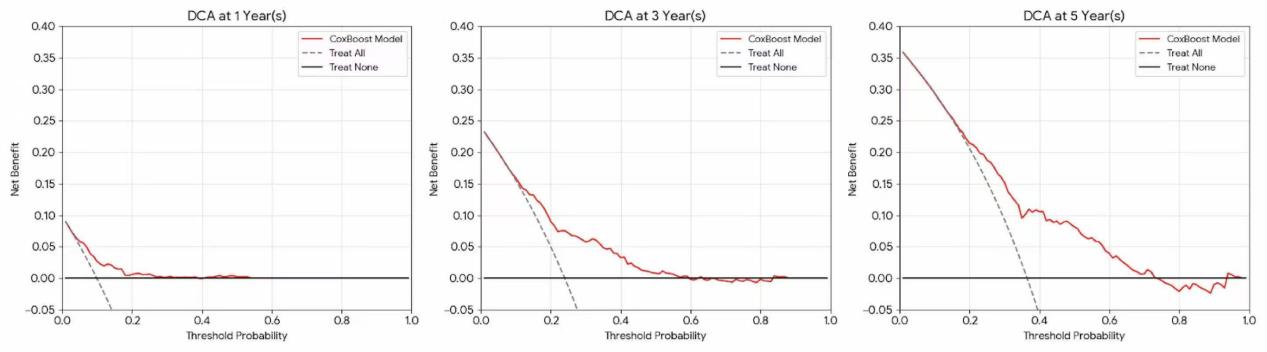


S2 Fig: **[Calibration curves of the prognostic model. ]**

[The plots illustrate the calibration of the model for predicting 1-, 3-, and 5-year overall survival (OS) in the TCGA-KIRC cohort. The x-axis represents the nomogram-predicted survival probability, and the y-axis represents the actual observed survival proportion. The gray diagonal dashed line represents the ideal prediction (perfect calibration). The close alignment of the model's performance lines with the diagonal indicates robust predictive accuracy.]
